# Experimental conditions affect the outcome of *Plasmodium falciparum *platelet-mediated clumping assays

**DOI:** 10.1186/1475-2875-7-243

**Published:** 2008-11-24

**Authors:** Mònica Arman, J Alexandra Rowe

**Affiliations:** 1 Institute of Immunology and Infection Research, School of Biological Sciences, University of Edinburgh, West Mains Rd, Edinburgh, EH9 3JT, UK

## Abstract

**Background:**

Platelet-mediated clumping of *Plasmodium falciparum*-infected erythrocytes (IE) is a parasite adhesion phenotype that has been associated with severe malaria in some, but not all, field isolate studies. A variety of experimental conditions have been used to study clumping *in vitro*, with substantial differences in parasitaemia (Pt), haematocrit (Ht), and time of reaction between studies. It is unknown whether these experimental variables affect the outcome of parasite clumping assays.

**Methods:**

The effects of Pt (1, 4 and 12%), Ht (2, 5 and 10%) and time (15 min, 30 min, 1 h, 2 h) on the clumping of *P. falciparum *clone HB3 were examined. The effects of platelet freshness and parasite maturity were also studied.

**Results:**

At low Ht (2%), the Pt of the culture has a large effect on clumping, with significantly higher clumping occurring at 12% Pt (mean 47% of IE in clumps) compared to 4% Pt (mean 26% IE in clumps) or 1% Pt (mean 7% IE in clumps) (ANOVA, p = 0.0004). Similarly, at low Pt (1%), the Ht of the culture has a large effect on clumping, with significantly higher clumping occurring at 10% Ht (mean 62% IE in clumps) compared to 5% Ht (mean 25% IE in clumps) or 2% Ht (mean 10% IE in clumps) (ANOVA, p = 0.0004). Combinations of high Ht and high Pt were impractical because of the difficulty assessing clumping in densely packed IE and the rapid formation of enormous clumps that could not be counted accurately. There was no significant difference in clumping when fresh platelets were used compared to platelets stored at 4°C for 10 days. Clumping was a property of mature pigmented-trophozoites and schizonts but not ring stage parasites.

**Conclusion:**

The Pt and Ht at which *in vitro *clumping assays are set up have a profound effect on the outcome. All previous field isolate studies on clumping and malaria severity suffer from potential problems in experimental design and methodology. Future studies of clumping should use standardized conditions and control for Pt, and should take into account the limitations and variability inherent in the assay.

## Background

*Plasmodium *parasites causing malaria are responsible for 300–500 million infections annually. Among the four species that infect humans, *Plasmodium falciparum *causes the majority of infections in Africa and is responsible for most severe disease and mortality [1,2]. The clinical outcome of *P. falciparum *infection is very diverse; some infections are asymptomatic, others develop into uncomplicated febrile disease, while 1–2% of infections cause severe complications, such as cerebral malaria or severe anaemia [3]. These clinical symptoms of malaria are attributed to the blood stage of the *P. falciparum *life cycle. During the intra-erythrocytic phase of the infection, *P. falciparum *has the unique ability to modify the surface of IE by inserting parasite-derived variant surface antigens with adhesive properties [4]. This results in erythrocytes infected by mature forms of the parasite (pigmented-trophozoites and schizonts) adhering to the microvascular endothelium of multiple organs and tissues and becoming sequestered from the peripheral circulation [4]. As a consequence of this sequestration, only IE with ring stage parasites are detected in peripheral blood. Although cytoadherence and sequestration of mature IE to the microvascular endothelium occurs in all infections, several adhesive phenotypes have been associated with severe pathological outcomes of malaria, such as the formation of rosettes (binding of IE to uninfected erythrocytes) [5], and the sequestration of IE in the microvasculature of the brain and the placenta [4].

**Figure 1:**
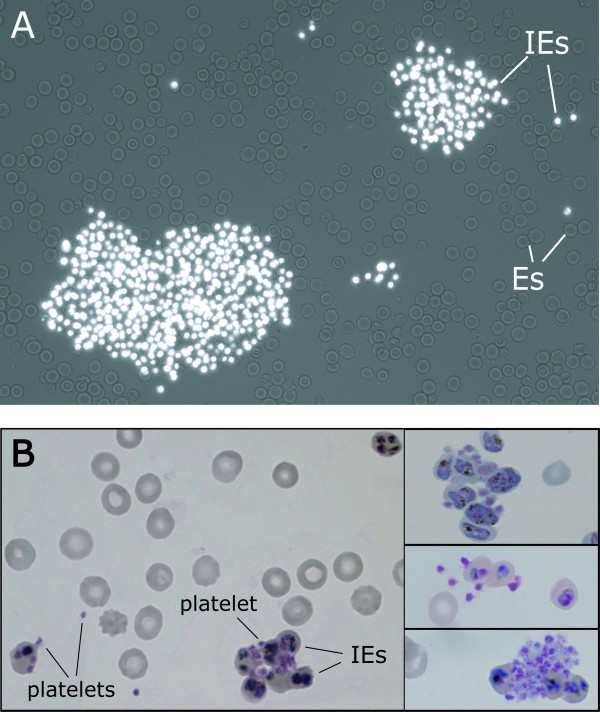
**Platelet-mediated clumps of *P. falciparum *infected erythrocytes (IE) detected by *in vitro *clumping assays**. A. Platelet-mediated clumps of infected erythrocytes (IEs) from *P. falciparum *clone HB3 viewed by ethidium bromide staining and fluoresence microscopy (400×) of a wet preparation. Uninfected erythrocytes (Es) present in the clumping assay are unstained. B. Platelet-mediated clumps of HB3 IEs observed by Giemsa-stained thin smears and light microscopy (1000×).

One of the most recent *P. falciparum *cytoadherence phenotypes to be described is the ability of IE to bind to platelets in suspension assays *in vitro *to form platelet-mediated clumps of infected cells [6] (Figure 1). This phenotype has been demonstrated in a wide range of *P. falciparum *laboratory strains and field isolates [6-10]. An association of the platelet-mediated clumping phenotype with severe or cerebral malaria has been reported after analysing field isolates from Kenya [6], Thailand [7] and Malawi [10]. However, a recent study in Mali showed a strong positive correlation between *P. falciparum *clumping *in vitro *and admission parasitaemia (percentage of erythrocytes infected with *P. falciparum *parasites), however, no significant association with severe malaria was seen [8]. In each of the above field isolate studies the clumping assay has been carried out in a different way. For example, the exact experimental conditions of haematocrit (Ht) (the percentage volume of the reaction occupied by erythrocytes), parasitaemia (Pt) and time of the clumping assay has varied between studies (Table 1).

**Table 1:** Summary of field isolate studies of clumping and malaria severity.

**Study**	**Site**	**Haematocrit**	**Parasitaemia**	**Time (min)**	**Significant association of clumping with:**
Pain *et al *2001 [6]	Kenya	5%	variable	15, 30, 60, 120	Severe malaria (impaired consciousness, respiratory distress, Hb < 5 g/dl)
Chotivanich *et al *2004 [7]	Thailand	1%	variable	15	Cerebral malaria and parasitaemia but not other forms of severe malaria (mostly multi-organ failure)
Arman *et al *2007 [8]	Mali	2%	variable	30	Parasitaemia but not severe disease
Wassmer *et al *2008 [10]	Malawi	5%	Adjusted to 10%	15, 30, 60, 120	Cerebral malaria and severe malarial anaemia

In order to determine whether the results of the Malian study [8], which differ from the others published [6,7,10], could be related to the assay conditions used, a detailed characterization of the *in vitro *platelet-mediated clumping assay was performed. The effects of Pt, Ht, and time of reaction on the assessment of clumping frequency were investigated, and the effects of platelet age (fresh versus stored) and parasite maturity were also assessed.

## Methods

### Parasite culture

The *P. falciparum *laboratory strains used in this study were HB3, IT/C10, 3D7 and Dd2. They were cultured in RPMI 1640 containing 2 mM glutamine, 25 mM Hepes, 20 mM glucose and 25 μg/ml gentamicin, with the pH adjusted to 7.2–7.4, and supplemented with 10% pooled human sera (complete RPMI). Cultures were set up at 1% haematocrit with blood group O erythrocytes and incubated at 37°C with 3% CO_2_, 1% O_2_, and 96% N_2_. Cultures were synchronized by sorbitol treatment as previously described [11]. The health and maturity of cultures were monitored by daily examination of thin blood smears stained with 10% Giemsa for 15–20 min. Cultures were tested regularly by PCR to exclude the possibility of mycoplasma contamination [12].

### Preparation of Platelet-Rich Plasma (PRP) and Platelet-Poor Plasma (PPP)

For all experiments, except the effect of platelet freshness on clumping (see below), whole blood was collected from O+ donors at the Scottish Blood Transfusion Service into citrate-phosphate-dextrose (CPD) and stored at 4°C before use. Blood used to obtain PRP was usually less than ten days old, although, very occasionally, it was up to two weeks old. PRP was isolated by centrifugation of whole blood for 10 min at 250 *g*. PPP was obtained by further centrifugation of the PRP fraction (3400 *g*, 30 min). The concentration of platelets was measured using a Neubauer haemocytometer. PRP and PPP were prepared freshly for each experiment.

### Platelet-mediated clumping assays and determination of clumping frequency

Clumping frequency was determined using the *in vitro *assay described by Pain *et al *[6], in which PRP is added to parasite cultures and mixed in suspension assays. Parasite cultures (mature pigmented-trophozoite stage) were stained with 25 μg/ml of ethidium bromide for 5 min at 37°C, and then used to set up mixtures of parasite culture and platelets at various Pt and Ht (see below). The cell suspensions in eppendorf tubes were gently rotated (10 rpm) at room temperature, and three wet preparations were made from each tube at each time point. Unless stated otherwise below, the time points used were 15 min, 30 min, 1 h and 2 h after the start of the assay. For each wet preparation, a 10 μl aliquot of culture suspension was placed on a clean microscope slide, covered with a 22 mm square coverslip and the edges sealed with nail polish to avoid drying out. Wet preparations were viewed on a fluroescence microscope (400× magnification) and at least 500 IE were counted and scored for clumping from each slide, with a clump being defined as three or more adherent IE. If a count of 500 IE was reached part way through a clump, counting was continued until all the cells in the clump had been counted. Clumping frequency was expressed as the percentage of IE in clumps out of the total number of IE counted. The approximate size of the clumps in the assays was recorded by estimating clump surface area by eye in comparison to the surface area of a clump of 50 IE. In all experiments, clumping frequency in PPP was used to control for the possibility of IE aggregation occurring without platelet-binding.

### Giemsa smears of clumping assays

To demonstrate the presence of platelets within the clumps of HB3 IE, a thin smear from an aliquot of the clumping assay was prepared and fixed with methanol prior to staining with 10% Giemsa for 30 min.

### Stability of clumps over time within a wet preparation

Two independent platelet-mediated clumping assays with HB3 were performed on different days, the first at 5% Ht, 1% Pt and the second at 10% Ht, 1% Pt. In each experiment, three sealed wet preparation slides were made after 30 minutes of the assay, and each of these wet preparations was counted repeatedly over a time period of five hours.

### Effect of Pt and Ht on clumping

Cell suspensions at different Pt or Ht were prepared from a single culture of HB3 and compared within the same experiment. Before setting up the experiment, the Pt of the culture was assessed by microscopy of a Giemsa-stained thin blood smear (400 cells counted in total). Then, an ethidium bromide-stained aliquot of the culture at 1% Ht was diluted with 0+ uninfected erythrocytes at 1% Ht in incomplete RPMI (as complete RPMI but without 10% serum) in order to achieve the desired final Pt (1%, 4% or 12%). Parasite samples were centrifuged and washed twice in incomplete RPMI. The packed cell volume of the samples was determined by comparison with tubes containing known volumes, and also by using a calibrated pipette to measure the volume recovered after resuspending the packed cell pellet with a known volume of incomplete RPMI. Platelet-mediated clumping reactions were started after resuspending the samples in an appropriate volume of incomplete RPMI plus PRP or PPP to obtain the final Ht to be tested. In all assays, the total volume of the clumping suspension was 500 μl. For the experiments performed at constant Ht, the same final platelet concentration (6.5 × 10^7 ^platelets/ml) was used in all the experiments, consistent with previous studies [6-8]. For the experiments performed at constant Pt the platelet concentrations were as follows: 10% Ht, 10 × 10^7 ^platelets/ml; 5% Ht, 5 × 10^7 ^platelets/ml and 2% Ht, 2 × 10^7 ^platelets/ml.

### Clumping in various laboratory strains

For the assessment of clumping frequency in C10, 3D7 and Dd2, the assays were set up at 10% Ht, 1% Pt with 4–10 × 10^7 ^platelets/ml. Wet preparations in triplicate were taken at 15 min, 30 min, 1 h and 2 h after the start of the assay. Clumping in HB3 (10% Ht, 1% Pt) was used as a positive control. Three replicate experiments were performed on different days with different PRP donors.

### Effect of platelet freshness on clumping

To analyse the effect of platelet storage on clumping, PRP samples obtained from fresh and stored blood from the same donor were compared in HB3 clumping reactions. A total of four healthy donors (who had not taken medication in the previous 14 days) were included in the study. In a first donation, blood was collected by forearm venepuncture into CPD anticoagulant and kept refrigerated (4°C). After seven days (donors 1 and 2) or ten days (donors 3 and 4) of storage, the same donors were bled again and the sample was kept at room temperature and used within 5 hours of venepuncture. PRP and PPP were obtained as described above. The HB3 clumping assays were set up at 10% Ht, 1% Pt with a final platelet concentration of 3.5 × 10^7 ^platelets/ml. Wet preparations in triplicate were taken at 15 min, 30 min and 1 h after the start of the assay. Assays with stored and fresh PPP were also performed in order to exclude the possibility of IE aggregation occurring without platelet-binding.

### Effect of anticoagulant on clumping

To analyse the effect of different anticoagulants on clumping, PRP samples from the same donor but containing different anticoagulants were compared in HB3 clumping assays. Two donors were bled and their blood samples immediately split into two tubes, one with CPD anticoagulant (Sigma C7165) and the second one with sodium citrate (Sigma S5770). All the samples were kept at room temperature and used within five hours. PRP aliquots were obtained from each blood sample as described above. The HB3 clumping assays were set up at 10% Ht 1% Pt with a final platelet concentration of 3.5 × 10^7 ^platelets/ml. Wet preparations in triplicate were taken at 15 min, 30 min and 1 h after the start of the assay.

### Effect of parasite maturity on clumping

To assess clumping throughout the asexual erythrocytic life cycle, aliquots of a synchronized HB3 culture were taken every eight hours and clumping assays were carried out at 10% Ht 1% Pt, with 6.5 × 10^7 ^platelets/ml. Wet preparations were taken in triplicate at 15 min, 30 min, and 1 h, as above. To complete the 48 h life cycle, seven assays were performed in which PRP was freshly prepared at each time point from aliquots of the same whole blood sample.

### Statistical analysis

Statview v5 software (SAS Institute Inc.) was used. For statistical comparisons of clumping under different conditions, the maximum mean clumping frequency from the four time points assessed for each condition was used, and maximum mean clumping frequencies were compared by ANOVA or Student's t test. A value of p < 0.05 was taken as statistically significant.

## Results and discussion

### Platelet-mediated clumping in *P. falciparum *laboratory strain HB3

The laboratory strain HB3 was assessed for the *in vitro *clumping phenotype. An ethidium-bromide-stained HB3 culture suspension (5% Ht, 5% Pt) was mixed with platelet-rich plasma to give a final platelet concentration of 3 × 10^7 ^platelets/ml and gently rotated at room temperature for 30 min. Examination of a wet preparation by fluorescence microscopy showed the presence of large clumps of IE (Figure 1A). Platelets are not detected by this procedure due to their lack of DNA, however, the involvement of platelets in the clumps of IE could be demonstrated using Giemsa-stained thin smears (Figure 1B). Two or more platelets could be seen binding between IE (Figure 1B, left and upper and middle right) and, occasionally, large platelet aggregates were seen within clumps (Figure 1B, bottom right). HB3, therefore, shows the platelet-mediated clumping phenotype *in vitro *and was used in subsequent experiments. HB3 may be particularly suitable for further laboratory investigations of clumping mechanisms because its full genome sequence is now available [13].

### Stability of clumps over time within a wet preparation

Because the planned experiments required assessment of clumping frequency in multiple wet preparations from assays set up under different conditions, and each wet preparation takes several minutes to count, it was necessary to determine whether the clumps were stable within the wet preparations over time. In the first experiment (5% Ht, 1% Pt), three wet preparation slides were prepared 30 min after the start of the assay and these slides were counted repeatedly at various time points after the preparation was made (from immediately to five hours later). There was only minor variation in the clumping frequency at repeated counts (slide 1: immediately (clumping frequency 39.7%), 20 min (48.3%), 35 min (37.7%), 265 min (26.8%), 280 min (41.4%) and 300 min (45.2%); slide 2: 55 min (31.0%), 100 min (22.0%) and 150 min (22.6%); slide 3: 60 min (29.6%), 125 min (28.2%) and 160 min (30.9%). Three slides prepared in a second independent experiment (10% Ht, 1% Pt, 30 min of clumping assay) confirmed that clumps are stable with time within a wet preparation (slide 1: 10 min (61.2%), 30 min (70.6%), 45 min (66.8%), 270 min (70.9%), 290 min (64.8%) and 310 min (72.2%); slide 2: 80 min (61.8%), 130 min (61.2%) and 180 min (68.2); slide 3: 90 min (57.0%), 140 min (63.6%) and 190 min (60.7%). Therefore, the comparison of multiple clumping assays set up simultaneously is feasible, as long as all slides are counted within at least five hours of preparation. Wet preparations of clumping cultures may be stable for longer time periods than five hours, however, this would require further experimental validation.

### Variation in clumping frequency between wet preparations

In the above experiments the variation in clumping frequency results derived from each individual wet preparation were compared. In experiment one, the clumping frequency counted from slide one was significantly higher than the other two slides (slide one mean clumping frequency 39.9% (SD 7.4), slide two mean 25.2% (SD 5.0), slide three mean 29.6% (SD 1.4), ANOVA p = 0.016). In experiment two, the differences between the mean clumping frequencies counted from the three independent wet preparations were less marked (slide one mean 67.8% (SD 4.3), slide two mean 63.8% (SD 3.9), slide three mean 60.4% (SD 3.3), ANOVA p = 0.073). Because of the variation in clumping frequency assessed from different wet preparations derived from the same assay tube, in all future experiments three wet preparations were counted for each condition and time point under study and the mean and standard deviation of the results from the three wet preparations are shown.

### Effect of parasitaemia and haematocrit on clumping frequency

#### Effect of parasitaemia on clumping frequency

**Figure 2:**
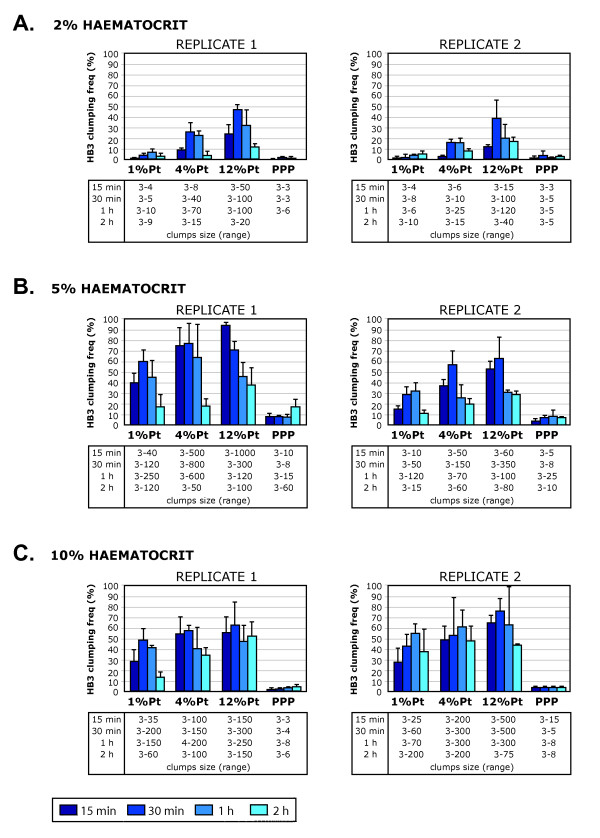
**Comparison of HB3 platelet-mediated clumping frequencies detected by *in vitro *assays performed at a constant haematocrit (Ht) but varying parasitaemia (Pt)**. A. *In vitro *platelet-mediated clumping assays were set up at 2% Ht and varying Pt (1%, 4% and 12%) and the clumping frequency (percentage of IE in clumps out of at least 500 IE counted) was assessed by fluorescence microscopy of wet preparations at four time points (15 min, 30 min, 1 h and 2 h). The mean and standard deviation of three wet preparations from each time point are shown, and the range of clump sizes (number of IE forming the clumps) detected in each assay and each time point is indicated. Two replicates of each experiment, done on different days and with platelet-rich plasma from different donors, are shown. Each experiment included a negative control performed with PPP (platelet-poor plasma) and 12% Pt to determine the potential for non-platelet mediated clumping. B. As above but at 5% Ht. C. As above but at 10% Ht.

Most of the clumping field studies published to date [6-8] have included a comparison of clumping frequencies amongst field isolates set up at a standardized Ht (1, 2 or 5%, Table 1) but with a wide range of different Pt, reflecting the varying admission Pt of malaria patients. In order to determine if this is an optimal experimental situation, the effect of Pt on clumping was investigated using the *P. falciparum *laboratory strain HB3. A total of six experiments were performed on different days where the Ht of the assays was fixed (2, 5 or 10%) and different Pt levels were compared within the experiment (1, 4, and 12%, Figure 2). In every experiment, all the assays were prepared from a single HB3 culture stained with ethidium bromide, and the clumping assay was allowed to proceed for four different time periods (15 min, 30 min, 1 h, and 2 h). Three wet preparations were made from each tube at each time point and clumping frequency was assessed by fluorescence microscopy.

At low Ht (2%), there was a marked effect of Pt on clumping, with significantly higher clumping frequencies being recorded for HB3 at 12% Pt than at 1% or 4% Pt (Figure 2A, ANOVA replicate 1 p = 0.0004, replicate 2 p = 0.017). At higher Ht (5% and 10%) the effect of Pt was less marked, and statistically significant differences between the maximum mean clumping frequencies at different Pts were not seen (Figure 2B, ANOVA replicate 1 p = 0.071, replicate 2 p = 0.10; Figure 2C, ANOVA replicate 1 p = 0.514, replicate 2 p = 0.185). However, assays combining high Ht (5 and 10%) and high Pt (4 and 12%) were problematic, because the clumps rapidly became huge in size (eg. Figure 1A) and unmanageable for accurate counting, even at the earliest time point (15 min) (Figure 2B and 2C). Another problem with assays performed at high Ht and high Pt was that the cells were so densely packed on the microscope slide, that for small clusters of IE, it was difficult to determine whether the cluster truly represented a platelet-mediated clump, or was just due to the IE sitting next to each other on the slide.

#### Effect of haematocrit on clumping frequency

**Figure 3:**
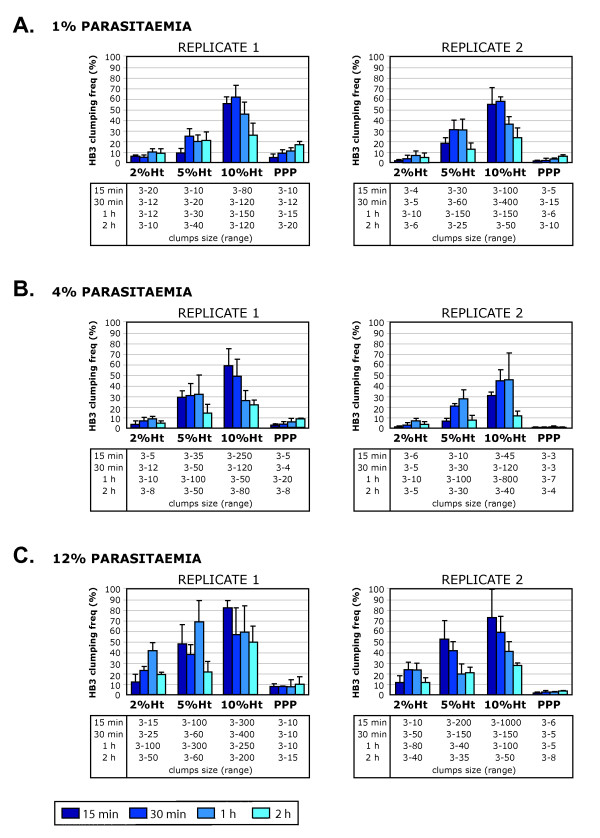
**Comparison of HB3 platelet-mediated clumping frequencies detected by *in vitro *assays performed at a constant parasitaemia (Pt) but varying haematocrit (Ht)**. A. *In vitro *platelet-mediated clumping assays were set up at 1% Pt and varying Ht (2%, 5% and 10%) and the clumping frequency (percentage of IE in clumps out of at least 500 IE counted) was assessed by fluorescence microscopy of wet preparations at four time points (15 min, 30 min, 1 h and 2 h). The mean and standard deviation of three wet preparations from each time point are shown, and the range of clump sizes (number of IE forming the clump) detected in each assay and each time point is indicated. Two replicates of each experiment, done on different days and with platelet-rich plasma from different donors, are shown. Each experiment included a negative control performed with PPP (platelet-poor plasma) and 10% Ht to determine the potential for non-platelet mediated clumping. B. As above but at 4% Pt. C. As above but at 12% Pt.

To further investigate the effect of experimental conditions on the clumping frequency detected from a single parasite culture, a second series of experiments were set up with HB3 by fixing the Pt and changing the Ht. This investigation was aimed at informing the most appropriate Ht assay conditions to use for field studies because parasite isolates collected from malaria patients show a wide range of Pt. A total of six experiments were performed on different days, and the Pt of the assays was fixed to 1, 4 or 12%, and different Ht conditions (2, 5 and 10%) were compared within the experiment (Figure 3).

Consistent with the results of Figure 2, it was found that when the Pt was low (1%), the Ht had a marked effect on the clumping frequency of the culture (Figure 3A), with a significantly higher maximum mean clumping frequency in assays set up at 10% Ht compared to those set up at 5% or 2% Ht (Figure 3A, ANOVA, replicate 1, p = 0.0004, replicate 2, p < 0.0001). At 4% Pt and 12% Pt, the effect of Ht was less marked, however some marginally significant differences were seen between assays set up at varying Ht (Figure 3B, ANOVA, replicate 1, p = 0.011, replicate 2, p = 0.061; Figure 3C, ANOVA, replicate 1, p = 0.021, replicate 2, p = 0.053). In all experiments, the highest maximum clumping frequency was detected at 10% Ht. This presumably reflects the increased likelihood at high Ht that IE with clumping potential will collide to bring about clumping. These results show that the Ht at which the clumping assay is performed has a substantial effect on the results obtained, especially in cultures with low Pt. As in the experiments described in Figure 2, it was found that assays combining high Pt (4 and 12%) and high Ht (5 and 10%) were problematic because of the formation of giant clumps involving hundreds of IE (Figures 1A and 3) and the difficulty assessing clumping in wet preparations containing densely packed IE.

The difficulty of accurately counting large clumps (> 30–50 IE) is a major problem in the clumping assay, and was the driving force behind the change from high Ht (5%) to low Ht conditions (1 or 2%) in some previous studies [7,8]. However, it is apparent from Figures 2 and 3 that the use of low Ht conditions can lead to an underestimation of the clumping frequency in assays set up at low Pt. The use of higher Ht conditions inevitably produces large clumps that are difficult to count (Figures 1, 2 and 3). It therefore seems that if the aim is to measure the maximum possible clumping frequency of a given parasite isolate, then it is necessary to accept the need to try and assess large clumps. The use of 10% Ht, 1% Pt represents one compromise solution, because even though large clumps are not avoided, the difficulty in assessing small cluster of IE on densely packed slides that occurs at higher Pt is prevented.

### Implications of results for previous field isolate studies investigating the relationship between clumping and malaria severity

The data shown in Figures 2 and 3 indicate that the Pt and Ht used to set up the *in vitro *clumping assay have a marked effect on the clumping frequency measured. This is important because in previous field isolate studies examining the relationship between clumping and severe malaria, parasite isolates with varying Pt (reflecting the varying admission Pt of the patients) have been studied. This is a particular problem because in most cases, the clinical categories under investigation have different mean Pt levels, with isolates from severe malaria patients tending to have higher Pt (and therefore higher clumping) than isolates from uncomplicated malaria controls. The effect of Pt on clumping therefore has the potential to bias the outcome of these disease association studies. Although statistical analysis can attempt to adjust results to account for Pt differences, this cannot entirely substitute for attempts to develop a better experimental design to avoid the problem of Pt bias.

One study attempted to standardize assays and overcome the problem of Pt bias by using gelatin purification of IE to adjust all Pts to 10% [10], with the assay being carried out at 5% Ht. Using this method, significantly higher clumping was seen in parasite isolates from severe malaria patients than in isolates from uncomplicated malaria controls [10], although the numbers in each disease category were small. This approach seems promising, however, as outlined above, it was found that assays combining high Ht (5 and 10%) and high Pt (4 and 12%) were problematic for two reasons. Firstly, with a highly clumping parasite such as HB3, the clumps rapidly become too huge to count accurately, even at the earliest time point (Figure 2B and 2C). These giant clumps tend to have a high three-dimensional component, so that even if an automated, computerized counting system from microscope images of the wet preparations could be developed, accurate assessment of the number of IE in giant clumps would still be problematic. Secondly, a problem of high Ht, high Pt assays that applies to all parasite isolates is that the cells are so densely packed on the microscope slide, that for small clusters of IE, it is difficult to determine whether the cluster truly represents a clump, or is just due to the IE sitting next to each other on the slide. For these reasons, accurate assessment of clumping will always be difficult in assays set up at high Ht and high Pt. An additional problem with the method of Wassmer *et al *[10], is that the gelatin purification of IE carried out to allow adjustment of all isolates to 10% Pt may cause the loss of IE of some phenotypes (eg. knobless or rosette-forming), resulting in a clumping frequency that does not truly reflect the clumping potential of the original isolate.

In another study [8], the problem of the Pt bias on clumping was avoided by comparing parasite isolates from two sets of patients with equally high mean Pts but differing levels of clinical disease (severe malaria patients compared to patients with uncomplicated high Pt malaria infections). There was no significant difference in clumping frequency between the parasite isolates from these two distinct clinical groups, suggesting that clumping is not specifically associated with severe malaria [8]. This is in contrast to the findings of Wassmer *et al *[10]. The differing conclusions of these two studies [8,10], which both attempted to control for the problem of Pt bias, indicates that further investigation of the association between clumping and malaria severity will be required.

One aspect of previously published field isolate studies that may be explained by the data shown here, is the strong correlation between Pt and clumping seen in the work of Chotivanich *et al *[7] and Arman *et al *[8], both of whom used assay conditions with low Ht (1 or 2%, Table 1) and varying Pt. As shown in Figures 2 and 3, at low Ht, the Pt at which the assay is set up has a significant effect on the clumping frequency obtained, whereas the Pt effect is less marked under the higher Ht conditions (5%) used by Pain *et al *[6] and Wassmer *et al *[10]. It seems likely that the studies using low Ht conditions [7,8] may have underestimated the true clumping frequency in field isolates with low Pt.

### Recommendations for future studies on clumping

It is apparent from the above discussion that all previous field isolate studies on clumping and malaria severity suffer from potential problems in experimental design and methodology. What then are the ideal conditions for assessing clumping frequency, and how can the effect of Pt be controlled for in field isolate studies comparing clumping in different patient groups? One possibility for avoiding Pt bias is to adjust all isolates to a standard Pt. The use of gelatin purification of IE and adjustment to high Pt (10%) as performed by Wassmer *et al *[10] brings with it the problems discussed above. However, one solution may be dilution of all samples to 1% Pt, combined with high Ht conditions (10%) to maximize clumping, while minimizing counting problems due to giant clumps and tightly packed IE on the slides. An alternative possibility would be to match severe malaria cases and uncomplicated malaria controls by Pt. There appears to be no perfect solution to the problem of how best to assess clumping, and the variability and limitations of the assay need to be appreciated, particularly when trying to compare results from different laboratories.

For experiments on laboratory parasite strains (for example investigating receptor-ligand interactions and the effect of antibodies or drugs on clumping), the use of 10% Ht, 1% Pt with a highly clumping strain such as HB3 provides conditions that give consistent results. However, other Ht and Pt combinations could be used providing they are kept constant within experiments.

#### Effect of time of the assay on assessment of clumpingfrequency

As can be observed in Figures 2 and 3, the time of the clumping assay that gave the highest clumping frequency varied among experiments, between 15 min and 1 h. Because of this variability, as suggested by Pain *et al *[6], multiple time points should be assessed in clumping experiments whenever possible. This may be especially important for field studies assessing isolates with a wide range of clumping frequencies in a single experiment. For well-characterized laboratory strains in which multiple experimental replicates can be performed, a reduction in the number of time points may be acceptable (eg. for HB3, the results shown here would not differ significantly if only the 30 min time point were studied).

One notable feature of the experiments shown in Figures 2 and 3 was that the clumping frequency was often lower at two hours compared to earlier time points. This suggests either that the clumps fall apart with time, or that the clumps become so huge that they cannot easily be pipetted and may become stuck to the walls of the tube. In some assays, especially those done at high Ht and high Pt, the wall and lid of the tube was often covered by dark red areas. When these areas were cleaned with a small volume of RPMI media and the recovered volume observed in a wet preparation, a large number of giant clumps were observed, suggesting that it is the formation of enormous clumps that is responsible for the apparent reduction in clumping frequency at two hours.

The difficulty in assessing huge clumps means that it is very difficult to get an accurate assessment of the true maximum clumping frequency of any parasite field isolate or laboratory strain. This limitation of the suspension assay suggests that it may be worthwhile to pursue alternative approaches to analysing the ability of IE to interact with platelets, such as the use of plate binding assays under static or flow conditions [14-16]. It is currently unclear whether platelet-mediated clumping measured in suspension assays and platelet-binding by IE measured in plate assays are identical phenotypes. It is also unclear how the ability to form platelet-mediated clumps in suspension assays *in vitro *relates to *in vivo *properties. It seems unlikely that giant clumps such as those shown in Figure 1A form *in vivo *because mature-IE would be expected to be sequestered, not floating free in suspension as in the *in vitro *clumping assay. However, it does seem plausible that, as suggested by Wassmer *et al *[17], platelet-binding to IE might promote cytoadherence and sequestration *in vivo*, and could target binding to endothelial beds not expressing adhesion receptors, such as CD36 [17].

The marked effect of assay conditions on clumping frequency is in contrast to another *P. falciparum *adhesion phenotype studied in suspension assays – the formation of rosettes by uninfected erythrocytes binding to IE. For rosetting, a phenotype that occurs spontaneously with mature pigmented-trophozoites and schizonts in culture, the experimental conditions of Ht, Pt and time of assay do not affect the final rosette frequency measured [18,19]. The variability inherent in the clumping assay will mean that careful standardization of methods will be required to allow useful comparison of data from different studies and different laboratories, making clumping a particularly challenging phenotype to investigate.

### Comparison of the clumping frequency among *P. falciparum *laboratory strains HB3, IT/C10, 3D7 and Dd2

**Figure 4:**
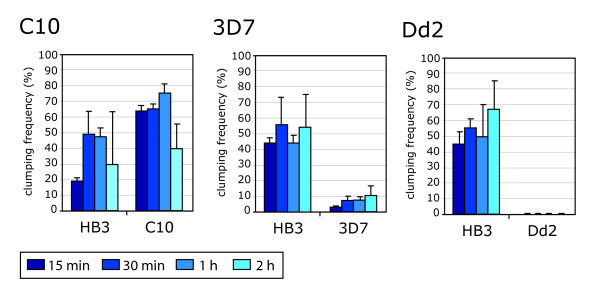
**Comparison of platelet-mediated clumping frequencies among laboratory strains of *P. falciparum***. The platelet-mediated clumping phenotype was analysed in the *P. falciparum *laboratory strains C10, 3D7 and Dd2, and their clumping frequencies compared to HB3. All the assays were performed at 10% Ht and 1% Pt. Three replicates of each experiment, done on different days and with platelet-rich plasma from different donors were performed and gave very similar results, therefore a representative experiment for each strain is shown. The clumping frequency values and ranges were obtained as described for Figure 2. Clumping frequency values in the graphs are the mean and standard deviation of three wet preparations counted from each time point.

The analysis of the platelet-mediated clumping phenotype in four laboratory strains with different genotypes was investigated to see whether the assay conditions that worked well for HB3 (10% Ht, 1% Pt, multiple time points), were also appropriate for other strains that may have different intrinsic clumping levels. It was found that IT/C10 had a high clumping frequency as described previously [6], 3D7 had low clumping frequency, and clumping was not detected in Dd2 (Figure 4). Highly similar results were seen in two further replicate experiments. The conditions of 10% Ht, 1% Pt and multiple time points therefore do allow differentiation between strains with different clumping levels, although from these data it cannot be guaranteed that these conditions are optimal for strains with low intrinsic clumping capacity. Further experiments were carried out using Dd2 to see if clumping could be detected by varying the conditions, however, clumping remained extremely low (less than 1% of IE in clumps) even after 2 h at 5% Ht, 12%Pt.

### Effect of platelet freshness on parasite clumping frequency

**Figure 5:**
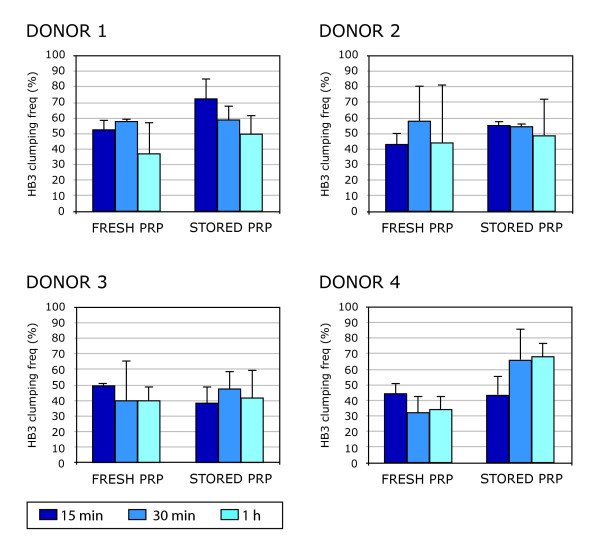
**Comparison of HB3 platelet-mediated clumping frequencies using fresh versus stored platelets**. Platelet-rich plasma (PRP) samples from fresh versus 4°C-stored blood from the same donors were compared for their ability to mediate clumping of HB3 parasites. For donors 1 and 2, stored PRP was obtained from 7 day-old whole blood samples and compared on the same day to PRP from fresh blood from the same donors. For donors 3 and 4, stored PRP was obtained from 10 day-old whole blood samples and compared on the same day to PRP from fresh blood from the same donors. In all cases the fresh PRP was kept at room temperature and used within five hours of venepuncture. *In vitro *platelet-mediated clumping reactions were performed at 10% Ht and 1% Pt. The clumping frequency is expressed as the percentage of IE in clumps out of at least 500 IE counted. Clumping frequency values in the graphs are the mean and standard deviation of three wet preparations counted from each time point.

Another important and neglected variable in the clumping assay is the handling of the platelets used to set up the assay. In particular, all previous studies have used platelets stored at 4°C for up to two weeks. Platelets become activated during prolonged storage at 4°C, leading to activation of signalling cascades, secretion of vasoactive compounds and alterations in the expression of cell surface adhesion molecules [20]. These processes obviously have the potential to influence the interaction between IE and platelets, and it is possible that clumping could be an artefact of *in vitro *platelet activation. The effect of platelet freshness was therefore investigated by comparing HB3 clumping frequency in the presence of either fresh platelets kept at room temperature and used within five hours of venepuncture, or platelets from the same donor that had been stored at 4°C for up to 10 days. Four different donors were tested and it was found that clumping occurred in both fresh and stored platelets (Figure 5). The mean maximum clumping frequency from the four donors using fresh PRP was 52.1% (SD 6.6), which did not differ significantly from the clumping frequency using stored PRP (mean 60.6%, SD 11.5, p = 0.28, paired t test). These results indicate that platelet binding to IE is a property of fresh platelets that is retained by old platelets, and is not merely an artefact resulting from platelet storage. Even though clumping occurs with both fresh and stored platelets, it is important that the functional effects of storage upon platelets are considered. In particular, functional studies of platelet responses to *P. falciparum *IE should be carried out using fresh platelets.

### Effect of anticoagulant on clumping

It is well documented that the nature of the anticoagulant used for blood collection can markedly affect platelet function. Sodium citrate is usually recommended in platelet research because it interferes less with platelet function than other anticoagulants such as heparin or EDTA [21,22]. In all the experiments described here, blood was collected into CPD (sodium citrate with the addition of phosphate buffer and dextrose to help to preserve erythrocytes). To exclude the possibility that the use of CPD is suboptimal, clumping was examined in fresh PRP from blood collected into CPD compared to blood collected from the same donor into sodium citrate. There was no statistically significant difference in maximum clumping frequency using PRP from blood collected into CPD compared to sodium citrate (donor 1: CPD, maximum mean clumping frequency 48.0%, SD 22.1; sodium citrate, mean 65.3%, SD 18.8, p = 0.35, unpaired t test. Donor 2: CPD, mean 64.3%, SD 29.6; sodium citrate mean 62.3%, SD 9.8, p = 0.91, unpaired t test).

### Effect of parasite maturity on platelet-mediated clumping

**Figure 6:**
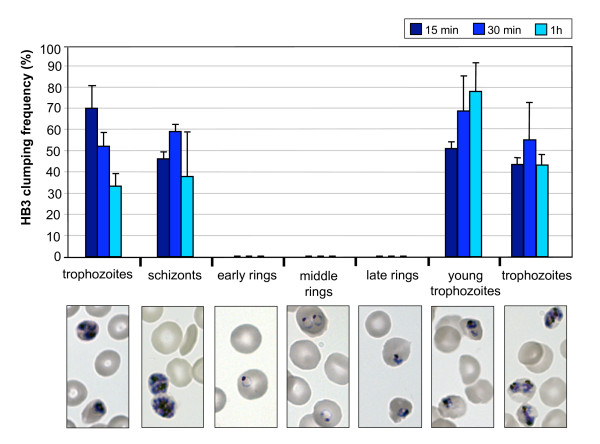
**Analysis of the platelet-mediated clumping phenotype throughout the different stages of the intraerythrocytic life cycle of *P. falciparum***. Aliquots of a synchronised HB3 culture were taken every eight hours and assessed for *in vitro *platelet-mediated clumping at 10% Ht and 1% Pt. Platelet-rich plasma from the same donor was used during the seven assays that were carried out to complete the life cycle. Representative pictures of Giemsa-stained thin smears of the HB3 culture aliquots analysed in each assay are shown below the graph. The clumping frequency is expressed as the percentage of IE in clumps out of at least 500 IE counted. Clumping frequency values in the graphs are the mean and standard deviation of three wet preparations counted from each time point. The graph shows the representative results of one out of three replicates of the experiment done with platelet-rich plasma from different donors.

In order to determine if clumping varies during the intraerythrocytic cycle of *P. falciparum*, clumping assays were performed every eight hours for an entire asexual cycle with synchronized HB3 parasites. As shown in Figure 6, only erythrocytes infected with mature forms of the parasite (pigmented-trophozoites and schizonts) were able to form clumps, and no clumps were detected using ring stage parasites. The maximum clumping frequency did not differ significantly between the different time points of pigmented-trophozoites and schizonts (ANOVA, p = 0.366). The timing of clumping over the asexual cycle is consistent with other *P. falciparum *adhesion phenotypes such as cytoadherence to endothelial cells and rosetting, which are also properties of pigmented-trophozoites and schizonts [23]. It is possible that the parasite variant surface antigen PfEMP1, which mediates other adhesion phenotypes of IE, may also be a parasite ligand for clumping, but this requires further investigation.

## Conclusion

The assessment of *P. falciparum *clumping is affected by the precise conditions used to set up the clumping assay *in vitro*, with Pt and Ht having a profound effect on the outcome of the assay. For field isolate studies, it is crucial that the effect of Pt on clumping is taken into account during experimental design, otherwise the higher Pt usually seen in parasite isolates from severe malaria patients compared to uncomplicated malaria controls could bias results. Possible solutions to the confounding effect of Pt on studies of clumping and malaria severity include adjustment of all isolates to a standardized Pt, or matching of cases and controls by Pt. For laboratory experiments on clumping, it is important that the limitations of the assay (eg. the difficulty in assessing the true maximum clumping frequency of a parasite because of the formation of giant clumps that cannot be counted accurately) are taken into account. Finally, the results shown here suggest that the low Ht assay conditions used in field isolate studies by Chotivanich *et al *[7] and Arman *et al *[8] may explain the strong correlation with Pt seen in these studies. However, the assay conditions alone cannot explain the variable association of clumping with severe malaria in different studies, which requires further investigation using carefully designed experiments and standardized techniques.

## Competing interests

The authors declare that they have no competing interests.

## Authors' contributions

MA designed and performed the experiments, and contributed to the analysis and interpretation of data. JAR conceived of the study, participated in the study design and interpretation of data, and performed the statistical analysis. Both authors wrote the paper and have read and approved the final manuscript.
